# Progress in Medicine

**Published:** 1899-12-16

**Authors:** 


### Progress in Medicine.

### NEUROLOGY.

(*Continued from page* 129.)

**Lumbar Puncture.**—Williamson,[18] in a critical review of the diagnostic and therapeutic value of lumbar puncture, says that the procedure is as follows: The patient is placed on the left side, and a needle is thrust in in the interval between the third and fourth lumbar vertebræ. In children the puncture is made in the middle line, in adults one c.m. to the side. The needle is pushed upwards and towards the middle line until the resistance is felt to cease owing to its entry into the subarachnoid space. Bacteriological examination of the fluid is of most importance; cover-glass preparations may be made, or cultivations may be made from the puncture fluid. In purulent meningitis, streptococci, staphylococci, the pneumococcus, and the meningococcus intracellularis have been detected. In tubercular meningitis the results have been more satisfactory than in other cases. The puncture fluid is usually clear, it may be turbid, and is seldom hæmorrhagic. Ten c.c. should be allowed to stand in a conical glass for 24 hours, and the fine clots which form examined for the bacilli. In 60 per cent. of verified cases the bacilli have been found. Hence, though a positive result is diagnostic, a negative one does not exclude the possibility of tubercular meningitis being present. Purulent fluid indicates a purulent inflammation. The repeated occurrence of hæmorrhagic fluid may be due to cerebral or spinal meningeal hæmorrhage, or to cerebral hæmorrhage bursting into the ventricle. Usually lumbar puncture has not caused any improvement in the various diseases in which it has been employed. But good results have been obtained in the somewhat indefinite class of diseases to which the term "serous meningitis of the ventricles," or "acute hydrocephalus," has been applied. Death has apparently resulted in one or two cases of cerebral tumour, particularly when situated in the posterior fossa of the skull. The chief value of lumbar puncture, therefore, is as a diagnostic agent, especially in suspected cases of tubercular meningitis, when a positive result enables a diagnosis to be made with certainty.

**Landry's Paralysis.**—Hayes[19] says that Landry's paralysis is probably an infectious disease of the nervous system; it is accompanied by an enlarged and softened spleen and enlarged mesenteric glands, and in some cases streptococci and staphylococci have been found. It lasts from a few days to fourteen weeks, and its typical condition is one of flaccid paralysis of all the extremities, with loss of reflexes, little disturbance of sensation, normal electrical reactions, uninvolved sphincters, rapid course, and universal fatality. The diagnosis is often most difficult, and one has to distinguish it from multiple neuritis, transverse myelitis, disseminated myelitis, and anterior poliomyelitis. In multiple neuritis there is generally a history of the ingestion of some poison or of some acute specific disease; though the onset is sudden the course is slow, sensibility is involved, electrical reactions are lost, atrophy is present, and it is generally without fatal issue. In transverse and disseminated myelitis sensibility and the sphincters are involved. In poliomyelitis the paralysis is limited to certain groups of muscles. Hayes suggests as a method of treatment a trial of anti-streptococcic serum, for Homen found, as a result of experimentally inoculating animals with these bacteria or their toxins, changes in the nervous system strikingly similar to those found in Landry's paralysis.

**Delirium Tremens.**—Jacobsen,[20] Villiers, and Hertz have during the year made important researches based on a large number of cases of delirium tremens. The age of the patients varied from 25 to 90 years, they were all chronic alcoholics, and the quantity consumed was equivalent to about half a pint of pure alcohol a day. Though fever was a common occurrence, yet it was not invariably present. The commonest visceral troubles accompanying the disease were catarrhal pneumonia and acute or subacute nephritis. Albuminuria was present in a varying proportion of cases—16 per cent. according to Villiers, 60 per cent. according to Jacobsen. Insomnia was very frequent. Of Villiers' patients 26 suffered from it for two days, 39 for three days, 22 for four days, and 10 for five days. From the above results there is every reason to suppose that delirium tremens is an acute auto-intoxication of the brain following the insufficient performance of renal functions as regards the elimination of autotoxic products as well as of alcohol. The special form which it takes—viz., hallucinatory and terrifying visions, tremor, wasting, and rapid, though temporary breakdown, is attributed to its occurring, as it almost invariably does, in persons debilitated by habits of previous alcoholic indulgence.

**Facial Diplegia.**—A case in which facial diplegia was present in association with other manifestations of polyneuritis has been recorded by Bernard and Braun.[21] A man, who had been exposed to rain for an hour and a half, had got wet through, and shortly afterwards began to be troubled by pains in the legs, followed by defective sensibility below the knees. On the sixth day of the disease examination of the patient revealed nearly absolute loss of power in the legs, with marked loss of sensibility in the same parts, and loss of tendon jerks. Total facial diplegia was present, the only movement possible being that of the lower jaw, the muscles of which are supplied by the fifth nerve. A little later the only response to faradism of the face was a slight flicker in the right orbicularis. About a month after the onset there was considerable improvement in walking, and the muscles of the left side of the face commenced to respond to faradism. Ten days later still the gait was normal, but the improvement of the facial paralysis was slow. The only etiological factor that could be found to explain the occurrence of the neuritis was exposure to cold and wet.

Siniger[22] also publishes records of two interesting cases of bilateral facial paralysis, probably due to peripheral neuritis. In the first case there was complete paralysis on both sides of the following cranial nerves: Third (except the pupil), fourth, sixth, and seventh, and incomplete affection of the eighth, eleventh, and twelfth. In the limbs the grasp was weak; there was slight dropping of both feet, and the knee-jerks were absent. Sensation and the action of the sphincters was normal. The affected muscles reached to faradism, with exception of the face muscles, which did not react. In the second case, two weeks after an attack of severe pain in the back, which was diagnosed as influenza, there was almost complete loss of power in the lower limbs, with severe burning pain and tenderness three days later. On admission the knee-jerks were absent, and a week later there was complete paralysis of both facial nerves. The paralysis was followed by wasting, with diminished reaction to faradism. The affection of the facial muscles was perfectly recovered from in both cases; in the former total recovery ensued, whilst in the latter case there was still some wasting of the leg muscles some months later.

**Peripheral Gouty Neuritis.**—Grube[23] points out that in sciatica of gouty origin the affection is rarely confined to the sciatic nerve; more frequently the crural nerve is simultaneously affected, and under certain circumstances the obturator nerve is also found diseased. The characteristics of the affection are: (1) Acute pains in the parts attacked—these pains both appear and disappear spontaneously; (2) great pain on pressure; (3) the muscles supplied by the nerves thus involved are usually in a state of atrophy, though of a mild type, and the response to the electric current is obviously diminished; (4) the knee-jerks are either reduced or absent; and (5) the well-known signs of paræsthesia and anæsthesia are almost regularly present. Neuritis of the brachial plexus, as a result of the uric acid diathesis, is even more frequently observed than of the sciatic nerve. As regards the treatment of such cases, besides the anti-gout remedies, the author has found beneficial the application of the constant current combined with the use of hot baths. It must be remembered, however, that the course of the affection is a very insidious one; indeed, several years may elapse before complete recovery ensues.

[18] Williamson, Med. Chron., June 1899. [19] Hayes, Med. Rec., May 27, 1899. [20] Jacobsen, Villiers, and Hertz, Jour. of Med. Sci., April, 1899. [21] Bernard and Braun, Lyon Med., 1898. [22] Siniger, Brit. Med. Jour., July 15, 1899. [23] Grube, Wien. Med. Woch., June 17, 1899.

